# The effect of a movable headrest in shoulder assist device for overhead work

**DOI:** 10.1017/wtc.2022.22

**Published:** 2022-10-03

**Authors:** Chiharu Ishii, Kanta Hirasawa

**Affiliations:** Department of Mechanical Engineering, Hosei University, Tokyo, Japan

**Keywords:** headrest, shoulder assist device, overhead work, %MVC, muscular stiffness

## Abstract

Recently, many kinds of shoulder-support exoskeletons have been developed and some of them are commercially available. However, to the best of our knowledge, shoulder-support exoskeletons that have neck-support mechanism have not been found. During the overhead work, physical strain is added to not only upper limb and shoulder but also neck of workers since the workers work keeping their face raised. Therefore, in this study, to reduce the physical strain on the neck during the overhead work, a movable headrest that can be attached to the shoulder assist device was developed, which has reclining and slide functions of a head. The main purpose of this article was to evaluate usefulness of the proposed movable headrest. To this end, measurements of electromyogram were carried out under simulating an overhead work activity, and the reduction effect for physical strain of the neck was compared among three types of headrests: (a) slide-type headrest which can slide the head backward and forward, (b) reclining-type headrest which can recline the head, and (c) reclining and slide-type headrest which can recline and slide the head. In addition, usefulness of the shoulder assist device with the proposed headrest was evaluated for a realistic overhead work activity through measurements of muscular stiffness of neck and shoulder. The experimental results showed that the existence of the headrest in the shoulder assist device is effective to reduce the physical strain to the workers, and that (c) reclining and slide-type headrest is the most effective among these three types of headrests.

## 1. Introduction

Recently, population is aging rapidly all over the world. According to the Statistics Bureau of Japan (2021), in 2020, the total population of Japan was 125.71 million, and the elderly population of 65 years old and over was 36.19 million, which means that the aging rate (proportion of the population of 65 years old or more) was 28.8%. Namely, currently one in four people in Japan is 65 years old or more. Actually, Japan is one of the countries with the highest aging rate in the world. On the other hand, in Japan, the number of the employed person of 65 years old or more was 5.7 million in 2010, but it increased up to 9.12 million in 2021 (Portal site of Official Statistics of Japan (e-Stat), 2021). From these facts, in order to reduce the physical strain in various occupations, the demand of the exoskeleton or wearable assist device increased. Thus, research and development of many kinds of exoskeletons and wearable assist devices were performed in various institutions, companies and organizations, and nowadays some of them resulted in practical use. Review on lower limb exoskeletons has been reported by Shi et al. (2019) and Pinto-Fernandez et al. (2020), and review on upper limb exoskeletons has been reported by Gull et al. (2020). Review of soft wearable robots has been summarized by Thalman and Artemiadis (2020).

### 1.1. Passive and active exoskeletons

Passive exoskeletons can reduce the physical strain added to workers in specific postures such as overhead work and stooping work (e.g., Chang et al., 2021 and Lamers and Zelik, 2021 for back-support exoskeleton). Evaluations of passive shoulder-support exoskeletons for overhead work have been conducted (e.g., Van Engelhoven et al., 2019 for “ShoulderX” (SuitX, Emeryville, CA), Vries et al. (2019) for “SkelEx” (Skelex, Rotterdam, The Netherlands) and Yamada et al. (2020) for “TasKi” (SoLARIS Inc., Tokyo, Japan). Active exoskeletons can enlarge the support range to the dynamic motion such as lifting work (e.g., Inoue and Noritsugu, 2018; Singer et al., 2020 for upper limb exoskeleton and Lazzaroni et al., 2021 for back-support exoskeleton). Missiroli et al. (2022) evaluated the effectiveness of a hybrid upper-limb occupational exoskeleton which combined a spring-loaded shoulder-support exoskeleton with an active elbow exosuit to support both shoulder and elbow flexion-extension in manual tasks. The results showed that the strain of the upper-limb muscles reduced by wearing the hybrid occupational exoskeleton.

### 1.2. Needs of ergonomic evaluation and in-field or industrial task study rather than laboratory-based study

As technology of exoskeletons progresses, evaluation of the safety and health effectiveness by wearing exoskeletons attracted attention of researchers. Thus, ergonomic evaluation of the exoskeletons and model-based analysis and optimization of the exoskeletons have been performed. Ergonomic evaluation of the wearable assist device for overhead work was executed by Rashedia et al. (2014), and reduction of physical strain on the upper limb and discomfort of low back were reported. Howard et al. (2020) discussed potential benefits and potential risks of upper limb exoskeletons and back-support exoskeletons, and indicated that more research is needed to develop safety standards for the safe use of exoskeletons in the workplace. For lifting a box, Marinou et al. (2021) investigated the effects of exoskeleton assistance and technical improvements in lifting technique based on the lumbar spine model, and revealed that the largest reduction of the risk of low-back injury occurs when both the exoskeleton and technical improvements are adopted.

Most recently, research trend of exoskeletons is changing from laboratory-based evaluations to in-field or industrial task evaluations that reflect the real-life working situation more. Crea et al. (2021) reviewed the effectiveness of occupational exoskeletons in laboratory and field studies, and proposed a roadmap to promote large-scale adoption of occupational exoskeletons. Bock et al. (2021) evaluated the effectiveness of two passive shoulder-support exoskeletons in in-field situation, and the results were compared. Then, the difference between laboratory-based evaluations and in-field evaluations of the exoskeletons was pointed out. Smets (2019) executed a field evaluation for overhead automotive assembly using the passive shoulder-support exoskeleton “EksoVest” (EksoBionics, Richmond, CA), and the results indicated that the exoskeleton may reduce some risk factors related to shoulder injuries. On the other hand, an effectiveness of an active shoulder-support exoskeleton was evaluated for overhead industrial tasks in terms of biosignals such as electromyography, heart rate, respiratory frequency, oxygen consumption by expired-gas analysis, and so on (Blanco et al., 2022). The results showed that the oxygen consumption, the heart rate and muscle activity reduced by wearing the active exoskeleton.

Field studies of passive back-support exoskeletons were carried out for workers in automobile manufacturing workplaces (Hensel and Keil, 2019) and for logistics workers (Siedl and Mara, 2021). An effectiveness of a powered back-support exoskeleton for aerial porters was examined for lifting and pushing tasks (Martin et al., 2022), and muscle activity and interaction with the user by wearing an active back-support exoskeleton were evaluated for the lifting, carrying, and lowering tasks in a manufacturing plant (Poliero et al., 2021).

### 1.3. Current neck assistive devices

In work-related musculoskeletal disorders, approximately 65% of complaints occur in the arms, back and neck or shoulder (Gatchel and Schultz, 2014). As described above, many kinds of exoskeletons to assist arms, back and shoulder have been developed to date and some of them are commercially available. However, assistive device for the neck has been barely discussed. Zhang and Agrawal (2018) developed an active neck brace to assist dropped head for patients such as amyotrophic lateral sclerosis. Yee and Kazerooni (2016) developed a passive neck orthosis to reduce neck pain for workers such as dentists by supporting workers’ heads during neck flexion. However, these assistive devices are for those whom their heads bent forward.

To the contrary, in order to support workers’ heads during overhead work, neck support may be available. However, if the neck support is used in the overhead work for a long time, neck will be sticky with perspiration. Thus, it is considered that use of the pure neck support is uncomfortable for workers in the workplace. Therefore, use of the headrest, which reduces the physical strain on the neck by supporting the head, is effective for the workers who keep their heads bent backward. During the overhead work, physical strain is added to not only upper limb and shoulder but also neck of workers since the workers work keeping their face raised. However, to the best of our knowledge, shoulder-support exoskeletons that have neck-support mechanisms have not been found.

### 1.4. Purpose of this study

Most of commercially available shoulder-support exoskeletons are passive, and aiming at low-pricing, a passive shoulder assist device was adopted in this study. Then, to reduce the physical strain on the neck during the overhead work, a movable headrest which can be attached to the shoulder assist device was developed. In the built headrest, the reclining of a head and slide of the head backward and forward are possible.

Then, main purpose of this study was to examine which kind of function is the most effective in the built movable headrest. To this end, measurements of electromyogram were carried out under simulating an overhead work activity, and the reduction effect for physical strain of the neck was compared among the headrest which can slide the head backward and forward, the headrest which can recline the head, and the headrest in which both the reclining and the slide of the head are possible.

Additional purpose of this study is to verify a usefulness of the total system, namely the shoulder assist device with the built headrest, for a realistic overhead work activity. To this end, measurements of muscular stiffness were carried out before and after simulating an overhead work closer to an actual work activity. Then, increasing rate of muscular stiffness under wearing the shoulder assist device with the headrest and that under not wearing the shoulder assist device with the headrest were compared.

### 1.5. Subjects

Although it may be often better to have many subjects in experiments, in this article, focusing on immediacy, five subjects (age: 22.8 ± 1.2 years old, body weight: 59.8 ± 5.3 kg and height: 1.69 ± 0.04 m) were enrolled in each experiment throughout this article. Even in small sample size of the subjects, the significant results have been reported in some literatures. For instance, Bock et al. (2021) enrolled four subjects, and Missiroli et al. (2022) enrolled six subjects in the experiments.

## 2. Assist device for overhead work

### 2.1. Overview of the shoulder assist device and its headrest for overhead work

#### 2.1.1. Shoulder assist device

**Figure 1.**
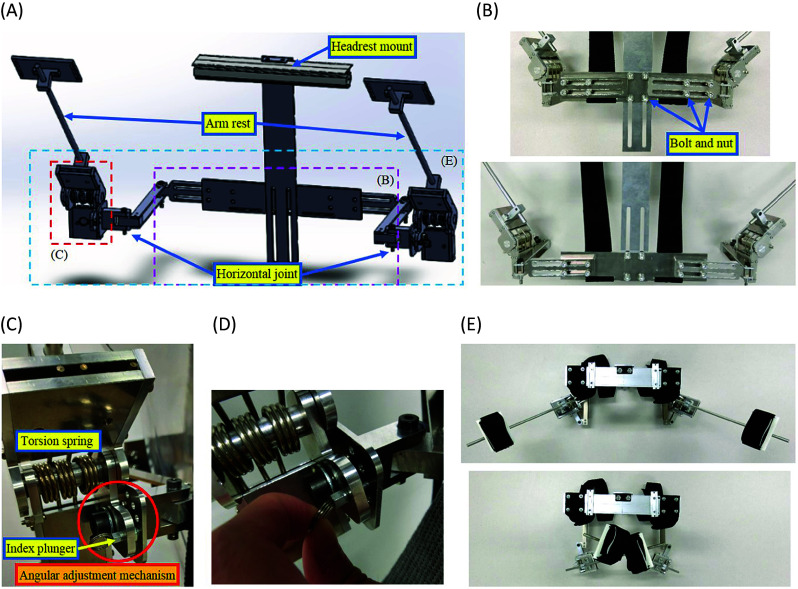
(a) 3D model of assist device for overhead work. (b) Size adjustment mechanism of height and width (upper: small size and lower: large size). (c) Support mechanism of the arms. (d) Angular adjustment mechanism of vertical flexion of the shoulder by index plunger. (e) Rotation of the horizontal joint from top view (upper: the maximum extension and lower: the maximum flexion).

In order to implement experimental works, a shoulder assist device for overhead work was designed and built. The three-dimensional model of the assist device for overhead work is shown in Figure 1a. The design is the same for left and right arms. For each arm, the device has two degrees-of-freedom, which are vertical flexion and horizontal flexion of the shoulder. A main body and frame arms are made of aluminum, and weight of the assist device is 2.645 kg.

The device is adjustable to adapt users of different sizes. As shown in Figure 1b, by fastening with bolt and nut, the height is 82 mm adjustable, and width is 60 mm adjustable for left and right, respectively. As shown in Figure 1c, three torsion springs are used to support each upper limb. Since the weight of human’s arm is about 1/17 of the body weight, the torsion spring was selected so that the load of 35–40 N can be supported (see Table A1 in Appendix A.1 for specifications of the torsion spring).

**Figure 2.**
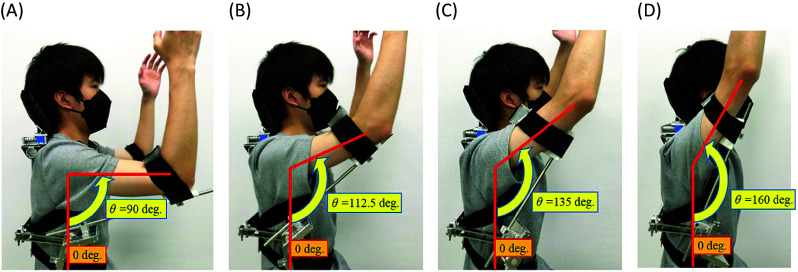
(a) The case where the vertical flexion angle 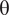 was fixed at 90°. (b) The case where the vertical flexion angle 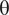 was fixed at 112.5°. (c) The case where the vertical flexion angle 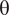 was fixed at 135°. (d) The case where the vertical flexion angle 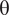 was fixed at 160°.

In the vertical plane, depending on the contents of work, multiple static poses where the arm can rest are achievable by changing the inserted hole of the index plunger in the angular adjustment mechanism shown in Figure 1c. As shown in Figure 1d, this manipulation can be done by the user themselves during the work. Around each static pose, the arm can move around locally due to the torsional springs. As shown in Figure 2, vertical flexion angle of the shoulder 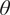 can be fixed at 90°, 112.5°, 135° and 160°, respectively.

**Figure 3.**
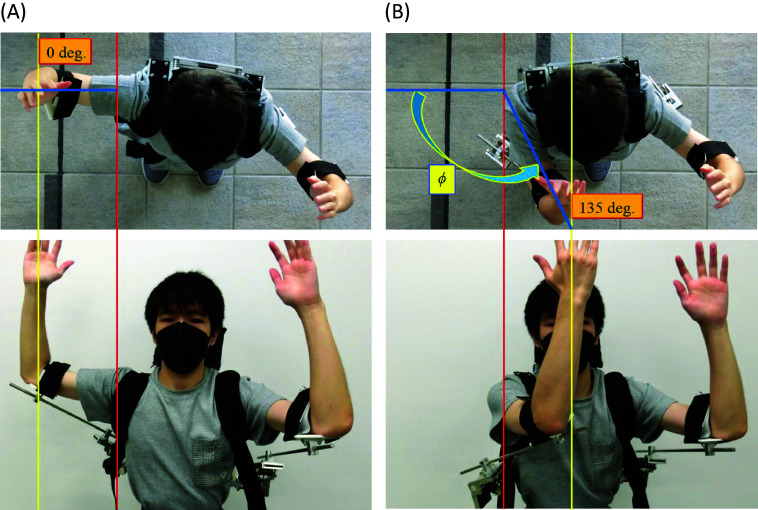
(a) Top view (upper) and front view (lower) when the horizontal flexion angle 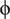 is 0° (right arm). (b) Top view (upper) and front view (lower) when the horizontal flexion angle 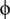 is 135° (right arm).

As shown in Figure 1e, the horizontal joint can rotate 135°. Thus, in the horizontal plane, the arm can rotate in 0° to 135° freely with the weight of the arm balanced due to the torsion springs. The case where the horizontal flexion angle of the shoulder 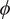 is 0° and 135° is shown in Figures 3a,b, respectively.

Since the elbow can be moved around freely, posture of adduction-abduction or medial-lateral rotation can be achieved for the fixed vertical flexion angle. Therefore, the developed shoulder assist device has sufficient degrees of freedom to perform typical overhead work such as fruit harvest and ceiling work.

#### 2.1.2. Movable headrest

**Figure 4.**
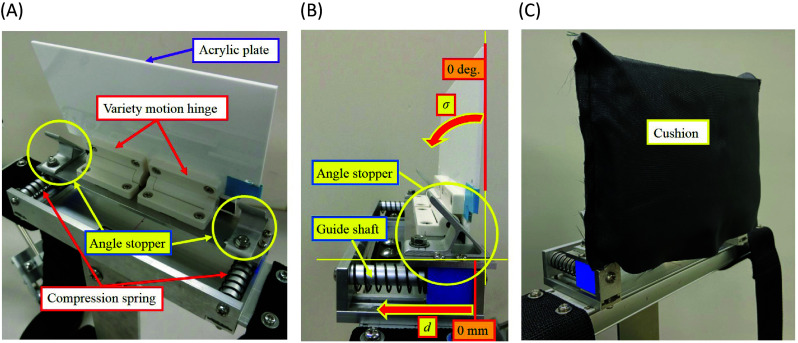
(a) Back view of the headrest for the shoulder assist device. (b) Side view of the headrest for the shoulder assist device when the angle stopper of 45° is used. (c) Overview of the built headrest for the shoulder assist device.

In order to reduce physical strain added to neck of the shoulder assist device wearers during the overhead work, a movable headrest that can be attached to the shoulder assist device was designed and built. The built headrest is shown in Figure 4. A main body is made of aluminum, and headrest parts are made of acrylic plate, and the acrylic plate is covered with cushion material as shown in Figure 4c. The dimensions of the headrest are 180 mm (width), 65 mm (depth), and 150 mm (height). The developed headrest is lightweight as 0.275 kg, and can be attached to the headrest mount of the shoulder assist device shown in Figure 1a.

**Figure 5.**
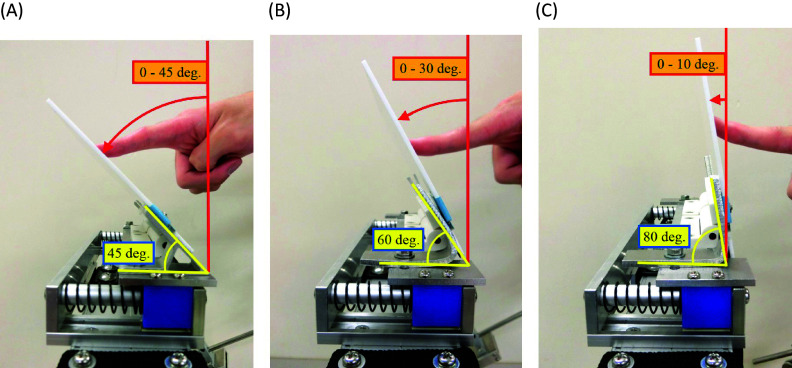
(a) The case where the angle stopper of 45° was attached. (b) The case where the angle stopper of 60° was attached. (c) The case where the angle stopper of 80° was attached.

The built headrest has reclining function of a head, and forward and backward slide function of a head. As for the reclining function, the acrylic plate is supported with two variety motion hinges in which a hinge and a torsion spring are unified, and the spring reaction torque is adjustable (see Table A2 in Appendix A.2 for specifications of the variety motion hinge). The reclinable angle 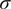 can be changed by replacing the angle stopper shown in Figures 4a,b. To investigate suitable reclining angle, three types of angle stopper (45°, 60° and 80°) were made. These angles were chosen to examine the difference of the physical strain to the neck every 15°–20°. When the angle stopper of 45° is attached, the reclinable angle is 0° to 45°, when the angle stopper of 60° is attached, the reclinable angle is 0° to 30°, and when the angle stopper of 80° is attached, the reclinable angle is 0° to 10°. These cases are shown in Figure 5, respectively. The angle stopper was changed manually by the experimenter at each experiment conducted later.

As for the slide function, as shown in Figures 4a,b, compression spring is surrounding the guide shaft at both sides of the headrest. When the head is leaned on the headrest, the acrylic plate slides horizontally along the guide shaft. Then, the reaction force of the compression spring supports the head. The displacement range *d* shown in Figure 4b is 0 to 27 mm. The stiffness of the compression spring was chosen by trial and error so that the acrylic plate slides smoothly and sufficient head support is obtained (see Table A3 in Appendix A.3 for specifications of the compression spring).

### 2.2. Fundamental experiments

To verify an effectiveness of the built shoulder assist device, experiments of measuring surface electromyogram (SEMG) during an overhead work activity were carried out under the conditions of wearing and not wearing the shoulder assist device, and to verify an efficacy of existence of the headrest in the shoulder assist device, experiments of measuring SEMG during an overhead work activity were carried out under the conditions of wearing the shoulder assist device with and without the headrest.

In this article, for experiments of measuring SEMG, to evaluate pure effectiveness of the developed shoulder assist device and the headrest, muscle activity was evaluated in static motion for the reason of exclusion of the additional force caused by users. On the other hand, muscular stiffness was evaluated through dynamic motion in Section 4.

#### 2.2.1. Experiments for the shoulder assist device

**Figure 6.**
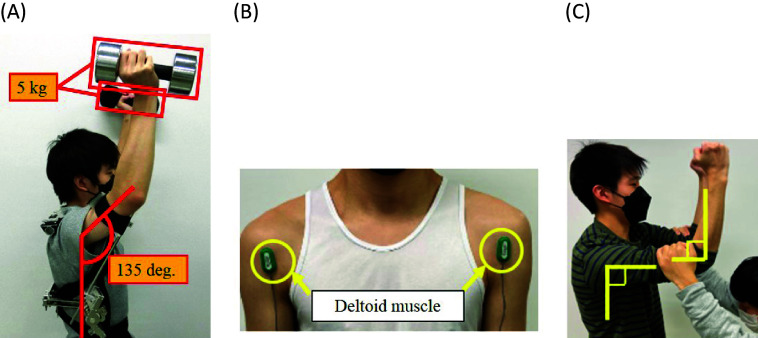
(a) Overhead work activity in the verification experiments of effectiveness of the shoulder assist device. (b) Front part of the deltoid muscle which is the measurement part of SEMG. (c) Measurement method of the maximum voluntary contraction (MVC).

As shown in Figure 6a, a subject maintains the vertical flexion angle of 135° and the horizontal flexion angle of 90°, and holds a 5 kg dumbbell by a hand of each side, respectively, for 10 s. Along the lines of the research in Yamada et al. (2020), the measurement part of SEMG was chosen as front part of the deltoid muscle shown in Figure 6b.

Throughout this article, SEMG was measured with sampling frequency 1 kHz using the EMG sensor SX230–1000 (Biometrics Ltd.), and integrated electromyogram (IEMG), which indicates the amount of muscle activity, was calculated by integrating the absolute value of the measured SEMG for a certain period. In this study, the integral period was set as 0.256 s as in the study of Morita et al. (2016). Then, in this article, based on the 100%MVC (maximum voluntary contraction) method, %MVC was calculated from the following equation:(1)

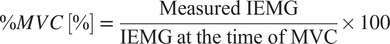



The measurement of MVC was referred to the Sorensen method (Kankaanpää et al., 1998). As shown in Figure 6c, the subject raised the upper limbs with standing posture, and resisted the load, which the experimenter added to the upper arms of the subject, by the maximum force with the posture maintained. The maximum value of SEMG at this time was measured, and it was regarded as the maximum voluntary contraction (MVC).

Once the EMG sensor is detached, it is difficult to attach the EMG sensor to the completely same placement, and a little difference of the placement of the EMG sensor affects the measurement results. Therefore, in subsequent experiments, the placement of the EMG sensor was fixed throughout each experiment for accurate comparison of the measured values.

Subjects are five healthy men described in Section 1.5, and the measurements were performed four times, respectively, to each subject. Then, mean and standard deviation of %MVC of the right and the left muscles were calculated for all the subjects. In addition, one-sided Welch’s *t* test was conducted for the mean values of %MVC under wearing the assist device and not wearing the assist device, in which the significance level was set at 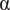 = 0.05 throughout this article. The results of the one-sided Welch’s *t* test are shown by the following symbols.

* indicates statistically significant difference with *p* < .05
** indicates statistically significant difference with *p* < .01
*** indicates statistically significant difference with *p* < .001
- *^n^* indicates no statistically significant difference with *p* > .05

#### 2.2.2. Experiments for the headrest

**Figure 7.**
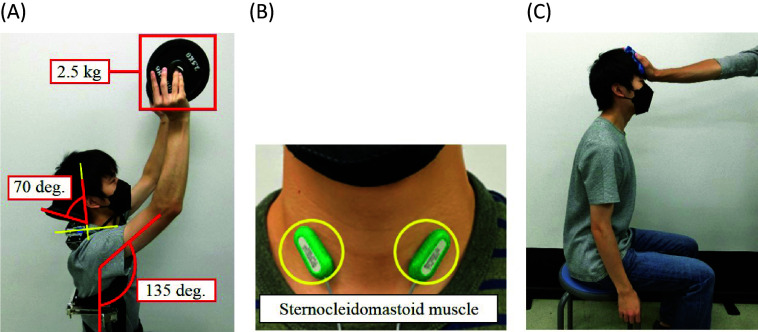
(a) Overhead work activity in the verification experiments of existence of the headrest. (b) Sternocleidomastoid muscle which is the measurement part of SEMG. (c) Measurement method of the maximum voluntary contraction (MVC).

As shown in Figure 7a, a subject maintains the vertical flexion angle of 135° and the horizontal flexion angle of 90° with the head leaning angle of 70°, and holds a 2.5 kg dumbbell by a hand of each side, respectively, for 10 s.

The sternocleidomastoid muscle plays an important role for rotation, flexion and extension of the neck. Therefore, the measurement part of SEMG was chosen as the sternocleidomastoid muscle shown in Figure 7b, and %MVC was calculated from equation (1). Similarly to Section 2.2.1, the measurement of MVC was referred to the Sorensen method (Kankaanpää et al., 1998). As shown in Figure 7c, the experimenter pushes the forehead of the sitting subject, and the subject pushes back by the maximum force of the neck with the posture maintained. The maximum value of SEMG at this time was measured, and it was regarded as the MVC.

Subjects are five healthy men described in Section 1.5, and the measurements were performed four times, respectively, to each subject. Then, mean and standard deviation of %MVC of the right and the left muscles were calculated for all the subjects. In addition, one-sided Welch’s *t* test was conducted for the mean values of %MVC under wearing the assist device with headrest and without headrest.

### 2.3. Experimental results

#### 2.3.1. Experimental results for the shoulder assist device

The measurement results are shown in Table 1.

**Table 1.** Mean of four times measurements ± standard deviation of %MVC

		The amount of muscle activity (%MVC) [%]					
Condition	Muscle	Subject 1	Subject 2	Subject 3	Subject 4	Subject 5	Average
Not wearing assist device	Left	43.3 ± 4.17	33.3 ± 5.73	38.2 ± 2.17	33.4 ± 3.15	49.9 ± 2.30	39.61
	Right	36.5 ± 3.31	35.3 ± 4.60	37.7 ± 2.66	34.1 ± 3.19	49.7 ± 2.51	38.65
Wearing assist device	Left	33.6 ± 2.33	19.5 ± 1.55	30.2 ± 3.58	22.6 ± 3.54	39.9 ± 1.59	29.16
	Right	25.6 ± 3.38	21.5 ± 2.96	27.0 ± 4.06	18.7 ± 2.03	32.4 ± 3.47	25.02

**Figure 8.**
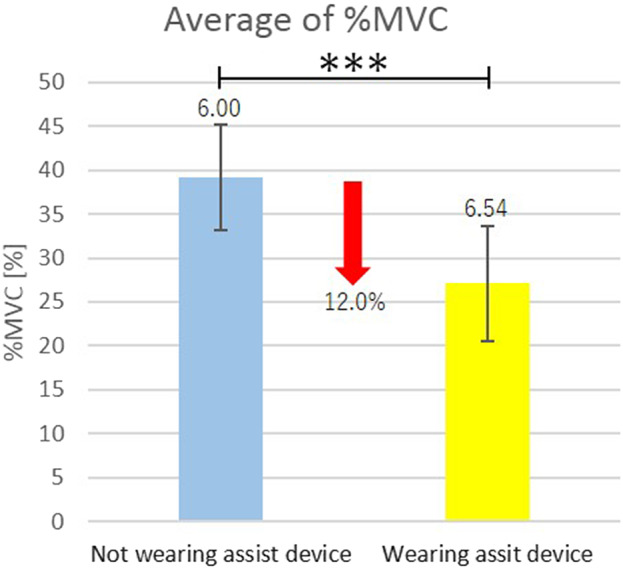
Efficacy of the shoulder assist device for overhead work.

Average of %MVC of the right and the left muscles for all the subjects are shown in Figure 8. In Figure 8, vertical segments with the value represent the standard deviation, and horizontal segment with *** connecting the bar graphs indicates statistically significant difference (*p* < .001) by the one-sided Welch’s *t* test.

From Table 1, for all the subjects, %MVC under wearing the shoulder assist device decreased as compared with that under not wearing the shoulder assist device for both the right and the left deltoid muscles. In addition, from Figure 8, a significant difference exists between the mean values of %MVC under wearing the shoulder assist device and the mean values of %MVC under not wearing the shoulder assist device. From these results, the effectiveness of the built shoulder assist device was verified as well as many of shoulder-support exoskeletons in the literatures mentioned in Section 1.

#### 2.3.2. Experimental results for the headrest

The measurement results are shown in Table 2.

**Table 2.** Mean of four times measurements ± standard deviation of %MVC

		The amount of muscle activity (%MVC) [%]					
Condition	Muscle	Subject 1	Subject 2	Subject 3	Subject 4	Subject 5	Average
Without headrest	Left	23.8 ± 2.09	31.0 ± 1.39	27.0 ± 0.39	30.6 ± 2.08	29.6 ± 3.28	28.41
	Right	23.9 ± 1.92	34.6 ± 1.64	28.4 ± 0.52	33.3 ± 3.05	21.1 ± 0.99	28.25
With headrest	Left	10.5 ± 0.46	17.5 ± 0.95	13.4 ± 0.67	13.8 ± 1.01	19.6 ± 1.32	14.97
	Right	13.2 ± 0.36	18.8 ± 0.87	13.2 ± 0.64	14.0 ± 0.74	15.5 ± 0.34	14.93

**Figure 9.**
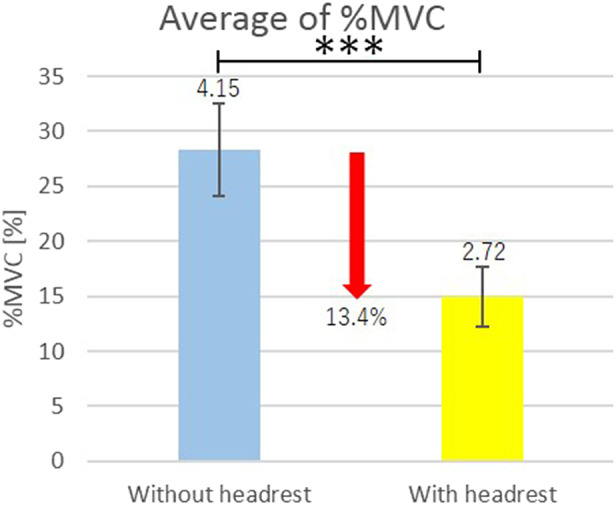
Efficacy of existence of the headrest in the shoulder assist device.

Average of %MVC of the right and the left muscles for all the subjects are shown in Figure 9. In Figure 9, vertical segments with the value represent the standard deviation, and horizontal segment with *** connecting the bar graphs indicates statistically significant difference (*p* < .001) by the one-sided Welch’s t test.

From Table 2, for all the subjects, %MVC under wearing the shoulder assist device with the headrest decreased as compared with that under wearing the shoulder assist device without the headrest for both the right and the left sternocleidomastoid muscles. In addition, from Figure 9, a significant difference exists between the mean values of %MVC under wearing the shoulder assist device with the headrest and the mean values of %MVC under wearing the shoulder assist device without the headrest. From these results, the efficacy of existence of the headrest in the shoulder assist device was verified.

## 3. Verification of efficacy of the headrest according to functional order

Now, since the efficacy of the headrest for reduction of the physical strain on the neck was confirmed, which kind of function is the most effective for the headrest of the shoulder assist device is examined based on the muscle activity calculated from measured SEMG.

The built headrest has both reclining function and slide function of the head. Therefore, the following three types of headrests are compared.

- Slide-type headrest: This is the case where only the slide function is available. The reclining angle of the headrest was fixed to a certain angle mechanically.
- Reclining-type headrest: This is the case where only the reclining function is available. The slide mechanism was locked mechanically.
- Reclining and slide-type headrest: This is the case where both the reclining function and the slide function are available. The wearer can lean his head on the headrest to a certain angle, and then the headrest moves backward.

### 3.1. Comparison between slide-type headrest and reclining-type headrest

#### 3.1.1. Experiments

**Figure 10.**
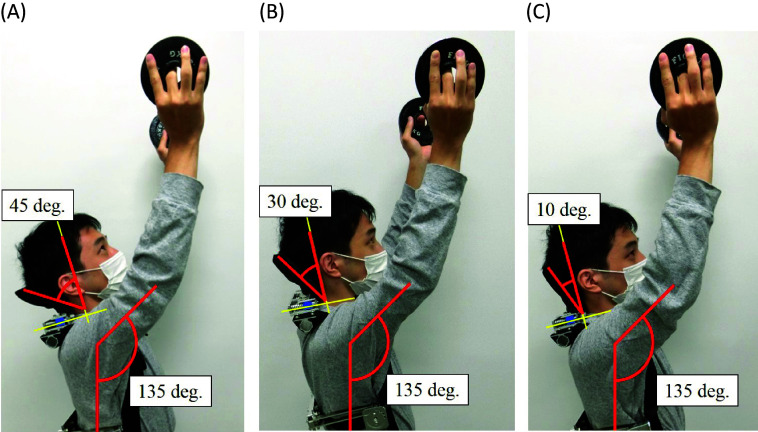
(a) Slide-type headrest by the head leaning angle of 45°. (b) Slide-type headrest by the head leaning angle of 30°. (c) Slide-type headrest by the head leaning angle of 10°.

First, experiments to compare (a) Slide-type headrest and (b) Reclining-type headrest were carried out. A subject wearing the shoulder assist device with the headrest maintains the vertical flexion angle of 135° and the horizontal flexion angle of 90°, and holds a 1.25 kg dumbbell by a hand of each side, respectively, for 10 s. Conditions of the headrest are as follows.

(a-1) Slide-45: In this case, the angle stopper of 45° was attached to the slide-type headrest, and the head leaning angle is fixed at 45° as shown in Figure 10a. Thus, the headrest moves backward maintaining the head leaning angle of 45°.

(a-2) Slide-30: In this case, the angle stopper of 60° was attached to the slide-type headrest, and the head leaning angle is fixed at 30° as shown in Figure 10b. Thus, the headrest moves backward maintaining the head leaning angle of 30°.

(a-3) Slide-10: In this case, the angle stopper of 80° was attached to the slide-type headrest, and the head leaning angle is fixed at 10° as shown in Figure 10c. Thus, the headrest moves backward maintaining the head leaning angle of 10°.

(b) Reclining: In this case, the slide mechanism was locked at zero displacement and the angle stopper of 45° was used in the reclining-type headrest. Thus, the subject can recline his head on the headrest up to the leaning angle of 45°.

The measurement part of SEMG is sternocleidomastoid muscle shown in Figure 7b. The measurement method of the MVC is the same as Section 2.2.2, and %MVC was calculated from equation (1). In addition, moving distance of the headrest was measured for three kinds of slide-type headrests ((a-1), (a-2) and (a-3)).

Subjects are five healthy men described in Section 1.5, and the measurements were performed four times, respectively, to each subject. Then, mean and standard deviation of %MVC of the right and the left muscles, and mean and standard deviation of moving distance of the headrest were calculated for all the subjects. In addition, one-sided Welch’s *t* test was conducted for the mean values of %MVC under wearing the assist device with slide-type headrest ((a-1), (a-2) and (a-3)) and reclining-type headrest.

#### 3.1.2. Experimental results

The measurement results of %MVC are shown in Table 3, and the measurement results of moving distance of the headrest are shown in Table 4.

**Table 3.** Mean of four times measurements ± standard deviation of %MVC

		The amount of muscle activity (%MVC) [%]					
Condition	Muscle	Subject 1	Subject 2	Subject 3	Subject 4	Subject 5	Average
Slide-45	Left	10.4 ± 0.79	15.7 ± 0.69	10.3 ± 0.87	14.7 ± 0.74	14.8 ± 1.10	13.19
	Right	11.7 ± 0.60	14.6 ± 1.78	13.0 ± 0.52	19.6 ± 0.43	18.2 ± 1.62	15.43
Slide-30	Left	11.1 ± 0.65	16.8 ± 1.35	11.5 ± 0.47	15.1 ± 0.94	15.1 ± 0.41	13.92
	Right	13.5 ± 0.61	15.6 ± 0.49	14.1 ± 0.39	20.4 ± 0.74	20.0 ± 1.29	16.71
Slide-10	Left	11.8 ± 1.55	15.0 ± 0.90	11.1 ± 0.48	14.9 ± 0.49	14.9 ± 0.82	13.53
	Right	13.6 ± 0.72	14.8 ± 1.03	13.9 ± 1.36	19.7 ± 0.77	20.2 ± 3.69	16.45
Reclining	Left	11.1 ± 0.64	16.7 ± 0.76	10.4 ± 0.80	15.3 ± 1.44	14.7 ± 0.38	13.65
	Right	12.9 ± 0.40	14.2 ± 0.72	13.1 ± 0.74	19.0 ± 0.44	19.8 ± 0.30	15.78

**Table 4.** Mean of four times measurements ± standard deviation of moving distance of headrest

	Moving distance *d* [mm]					
Condition	Subject 1	Subject 2	Subject 3	Subject 4	Subject 5	Average
Slide-45	10.3 ± 0.58	7.1 ± 0.37	8.3 ± 0.38	7.6 ± 0.25	9.4 ± 0.31	8.54
Slide-30	12.9 ± 0.80	8.9 ± 0.69	9.1 ± 0.27	7.6 ± 0.18	10.2 ± 0.38	9.74
Slide-10	15.1 ± 0.51	10.9 ± 0.29	9.6 ± 0.35	10.8 ± 0.15	13.0 ± 0.49	11.9

**Figure 11.**
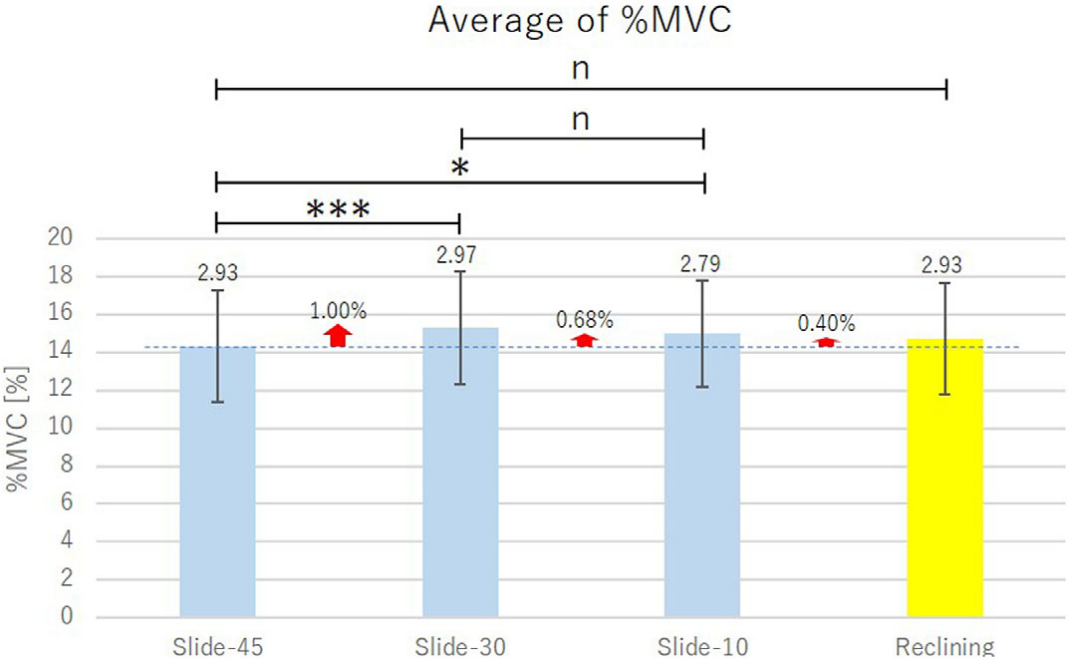
Comparison of slide-type headrest and reclining-type headrest.

Average of %MVC of the right and the left muscles for all the subjects are shown in Figure 11. In Figure 11, vertical segments with the value represent the standard deviation, horizontal segment with *** connecting the bar graphs indicates statistically significant difference (*p* < .001), horizontal segment with * connecting the bar graphs indicates statistically significant difference (*p* < .05) and horizontal segment with *^n^* connecting the bar graphs indicates no statistically significant difference (*p* > .05) by the one-sided Welch’s *t* test.

From Table 3 and Figure 11, for the value of %MVC, it seems that there is no big difference between the slide-type headrest and the reclining-type headrest. When Slide-30 and Slide-10 are compared with Slide-45, %MVC of Slide-45 is the smallest. Actually, a significant difference exists between the mean values of %MVC of Slide-45 and the mean values of %MVC of Slide-30 and Slide-10. However, no significant difference exists between the mean values of %MVC of Slide-30 and those of Slide-10, and between the mean values of %MVC of Slide-45 and those of Reclining. From Table 4, moving distance of the headrest of which the head leaning angle is small is larger. This is because when leaning the headrest with small head leaning angle, larger backward force occurs.

From these experimental results, it was verified that Slide-45 is the most effective among these four kinds of headrests ((a-1), (a-2), (a-3) and (b)) to reduce the physical strain added to sternocleidomastoid muscle.

### 3.2. Comparison between slide-type headrest and reclining and slide-type headrest

#### 3.2.1. Experiments

Second, since Slide-45 in (a) Slide-type headrest was most effective for supporting the neck, experiments to compare (a-1) Slide-45 and (c) Reclining and slide-type headrest were carried out. Experimental method is the same as Section 3.1.1. Conditions of the headrest are (a-1) and the following.

(c) Reclining-slide: In this case, both reclining and slide of the headrest are possible. The angle stopper of 45° was used in the headrest. Thus, the subject can recline his head on the headrest up to the leaning angle of 45°, and then the headrest moves backward.

The measurement methods are also same as Section 3.1.1. Then, mean and standard deviation of %MVC of the right and the left muscles, and mean and standard deviation of moving distance of the headrest were calculated for all the subjects. In addition, one-sided Welch’s *t* test was conducted for the mean values of %MVC under wearing the assist device with slide-type headrest (a-1) and reclining and slide-type headrest.

#### 3.2.2. Experimental results

The measurement results of %MVC are shown in Table 5, and the measurement results of moving distance of the headrest are shown in Table 6.

**Table 5.** Mean of four times measurements ± standard deviation of %MVC

		The amount of muscle activity (%MVC) [%]					
Condition	Muscle	Subject 1	Subject 2	Subject 3	Subject 4	Subject 5	Average
Slide-45	Left	10.4 ± 0.79	19.3 ± 1.45	20.4 ± 1.71	14.3 ± 1.50	10.7 ± 0.45	15.04
	Right	11.7 ± 0.60	12.8 ± 0.51	20.5 ± 1.02	11.5 ± 0.23	10.6 ± 0.43	13.41
Reclining-slide	Left	10.7 ± 0.82	16.8 ± 0.49	17.7 ± 0.85	8.14 ± 0.52	12.1 ± 0.87	13.10
	Right	11.2 ± 1.08	12.5 ± 0.37	17.5 ± 0.70	8.74 ± 0.52	10.8 ± 0.50	12.13

**Table 6.** Mean of four times measurements ± standard deviation of moving distance of headrest

	Moving distance *d* [mm]					
Condition	Subject 1	Subject 2	Subject 3	Subject 4	Subject 5	Average
Reclining-slide	9.8 ± 0.34	7.1 ± 0.23	7.9 ± 0.26	7.5 ± 0.22	8.9 ± 0.31	8.24

**Figure 12.**
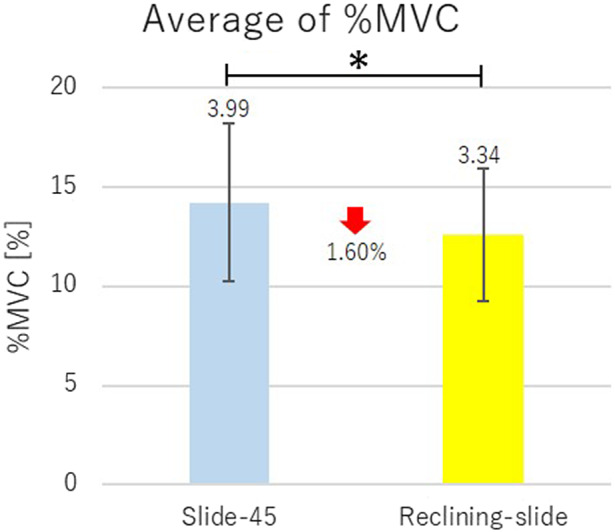
Comparison of slide-type headrest Slide-45 and reclining and slide-type headrest.

Average of %MVC of the right and the left muscles for all the subjects are shown in Figure 12. In Figure 12, vertical segments with the value represent the standard deviation, and horizontal segment with * connecting the bar graphs indicates statistically significant difference (*p* < .05) by the one-sided Welch’s *t* test.

From Table 5, when Reclining-slide is compared with Slide-45, decrease of %MVC was seen in Reclining-slide for either or both of the right and the left sternocleidomastoid muscles except for Subject 5. Actually, from Figure 12, a significant difference exists between the mean values of %MVC of Reclining-slide and the mean values of %MVC of Slide-45. From Table 4 and 6, it can be said that the moving distance of Reclining-slide and Slide-45 is in the same level.

From these experimental results, it was verified that Reclining-slide is the most effective among three types of headrests ((a), (b) and (c)) to reduce the physical strain added to sternocleidomastoid muscle.

#### 3.2.3. Discussion

The difference between Slide-45 and Reclining-slide is interpreted as follows. In Slide-45, the head leaning angle is fixed at 45°. On the other hand, in Reclining-slide, the head leaning angle can be altered between 0° to 45°. Therefore, Reclining-slide has high degree of freedom as compared with Slide-45. In addition, in Reclining-slide, the head of the wearer constantly receives the reaction force by torsion springs in the variety motion hinges; thereby, the wearer’s neck is much supported. These are the reasons for why Reclining-slide is superior to Slide-45.

### 3.3. Verification of effect of the headrest on deltoid muscle

#### 3.3.1. Experiments

From the above verifications, the effectiveness of the headrest of the shoulder assist device on sternocleidomastoid muscle was confirmed. Here, the effectiveness of the headrest of the shoulder assist device on other muscle is examined. To this end, experiments of measuring SEMG of front part of the deltoid muscle shown in Figure 6b were carried out during an overhead work activity under the conditions of wearing the shoulder assist device with and without the headrest.

A subject maintains the vertical flexion angle of 135° and the horizontal flexion angle of 90°, and holds a 5 kg dumbbell by a hand of each side, respectively, for 10 s. The most effective (c) Reclining-slide was used as the headrest. The measurement method of the MVC is the same as Section 2.2.1, and %MVC was calculated from equation (1).

Subjects are five healthy men described in Section 1.5, and the measurements were performed four times, respectively, to each subject. Then, mean and standard deviation of %MVC of the right and the left muscles were calculated for all the subjects. In addition, one-sided Welch’s *t* test was conducted for the mean values of %MVC under wearing the assist device with headrest and without headrest.

#### 3.3.2. Experimental results

The measurement results are shown in Table 7.

**Table 7.** Mean of four times measurements ± standard deviation of %MVC

		The amount of muscle activity (%MVC) [%]					
Condition	Muscle	Subject 1	Subject 2	Subject 3	Subject 4	Subject 5	Average
Without headrest	Left	30.2 ± 3.58	33.6 ± 2.33	19.5 ± 1.55	22.6 ± 3.54	39.9 ± 1.59	29.16
	Right	27.0 ± 4.06	25.6 ± 3.38	21.5 ± 2.96	18.7 ± 2.03	32.4 ± 3.47	25.02
With headrest	Left	26.7 ± 0.67	33.8 ± 2.57	16.8 ± 3.90	25.6 ± 0.50	26.7 ± 5.20	25.89
	Right	24.3 ± 1.81	24.3 ± 4.25	16.2 ± 2.57	16.8 ± 2.42	21.4 ± 2.24	20.58

**Figure 13.**
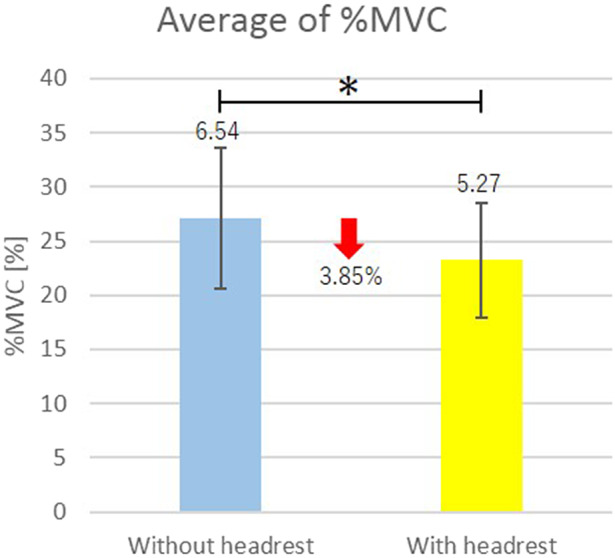
Effect of the headrest of the shoulder assist device on deltoid muscle.

Average of %MVC of the right and the left muscles for all the subjects are shown in Figure 13. In Figure 13, vertical segments with the value represent the standard deviation, and horizontal segment with * connecting the bar graphs indicates statistically significant difference (*p* < .05) by the one-sided Welch’s *t* test.

From Table 7, for all subjects, decrease of %MVC was seen under wearing the shoulder assist device with the headrest for either or both of the right and the left deltoid muscles. In addition, from Figure 13, a significant difference exists between the mean values of %MVC under wearing the shoulder assist device with the headrest and the mean values of %MVC under wearing the shoulder assist device without the headrest. From these results, the effectiveness of the headrest of the shoulder assist device on deltoid muscle was verified.

## 4. Verification of usefulness of the movable headrest for overhead work

Finally, a usefulness of the shoulder assist device with the headrest when performing an overhead work closer to an actual work activity is examined. However, there is a technical difficulty to measure EMG for long time work activity due to a limitation of memory space of the measurement device used in this study. Therefore, for a comparatively long time work activity, experiments of measuring muscular stiffness are conducted using a muscle hardness tester. It is known that when the muscle stiffness becomes large, illness such as stiff shoulders occurs (Uchida et al., 2011).

### 4.1. Experiments of measuring muscular stiffness

**Figure 14.**
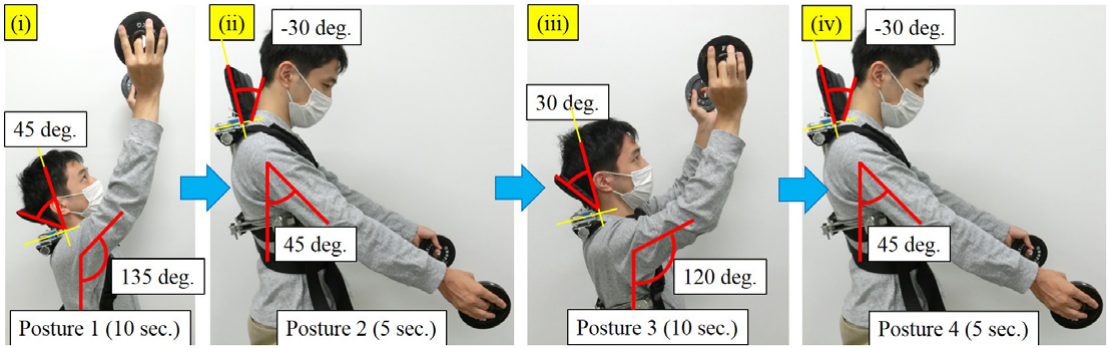
Simulated realistic overhead work activity in experiments.

A realistic overhead work activity performed in the experiments is shown in Figure 14, and illustrated as follows. This overhead activity simulates harvesting fruits and putting them in a basket.

Throughout the experiment, a subject wears the shoulder assist device with the headrest, and holds a 1.25 kg dumbbell by a hand of each side, respectively.

Posture 1: The subject maintains the vertical flexion angle of 135° and the horizontal flexion angle of 90° with the head leaning angle of 45°, and maintains this posture for 10 s.

Posture 2: The subject maintains the vertical flexion angle of 45° and the horizontal flexion angle of 90° with the head leaning angle of −30°, and maintains this posture for 5 s.

Posture 3: The subject maintains the vertical flexion angle of 120° and the horizontal flexion angle of 90° with the head leaning angle of 30°, and maintains this posture for 10 s.

Posture 4: The subject maintains the vertical flexion angle of 45° and the horizontal flexion angle of 90° with the head leaning angle of −30°, and maintains this posture for 5 s.

The subject performs the above four postures in turn, and then takes a rest for 30 s. This cycle is repeated 10 times, (namely, for 10 min) continuously. Conditions of the experiments are as follows.

- No device: The subject wears no assist device.
- Device with Slide-45: The subject wears the shoulder assist device with the slide-type headrest (a-1) Slide-45.
- Device with Reclining: The subject wears the shoulder assist device with the reclining-type headrest (b) Reclining.
- Device with Reclining-slide: The subject wears the shoulder assist device with the reclining and slide-type headrest (c) Reclining-slide.

**Figure 15.**
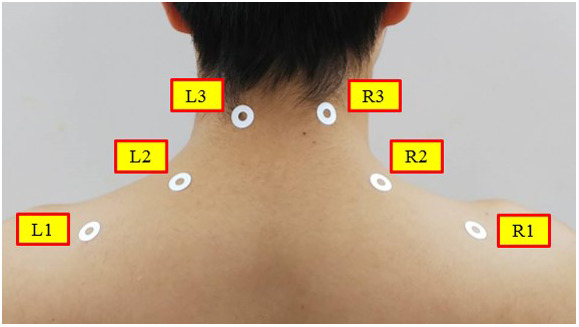
Measurement positions of muscular stiffness.

The measurement positions of muscular stiffness 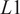 , 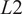 , 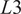 , 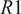 , 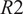 and 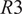 were determined according to Uchida et al. (2011), which are on the trapezius muscle, splenius capitis muscle and splenius cervicis muscle as shown in Figure 15. Therefore, an effect of the assist device on the muscles at the back of the upper trunk and the neck is evaluated in the experiments.

Subjects are five healthy men described in Section 1.5. Since the values of muscular stiffness differ for each subject, variations of the muscular stiffness before and after overhead work activity were employed for evaluation. For this reason, muscular stiffnesses before performing 10 min overhead activity and after performing 10 min overhead activity were measured for each subject using the muscle hardness tester NEUTONE TDM-NA1 (TRY-ALL Co.). In the preliminary experiment executed in advance, the difference of the muscular stiffness was observed 10 min later after the overhead work activity began. Thus, time of overhead work activity was determined as 10 min in this study.

In addition, there exists dispersion in the measured values of the muscular stiffness. For this reason, the muscular stiffnesses before and after performing 10 min overhead activity were measured five times, respectively, to each subject. Then, the maximum and the minimum values were removed from five measurements, respectively, and the mean of the remaining three measurements was adopted as the measured value of muscular stiffness. Then, mean and standard deviation of the muscular stiffness of each measurement position for every experimental condition were calculated for all the subjects.

### 4.2. Experimental results

The results of the muscular stiffness before and after performing 10 min overhead activity are shown in Table 8.

**Table 8.** Mean of three measurements ± standard deviation of muscular stiffness

		The muscular stiffness [−]							
		No device		Device with Slide-45		Device with Reclining		Device with Reclining-slide	
Subject	Position	Before	After	Before	After	Before	After	Before	After
Subject 1	L1	38.8 ± 0.97	41.4 ± 0.74	41.4 ± 0.87	42.1 ± 0.83	35.5 ± 1.00	37.5 ± 1.28	41.0 ± 0.38	41.4 ± 0.34
	L2	28.7 ± 0.33	31.1 ± 1.84	30.3 ± 1.88	31.9 ± 0.26	25.3 ± 0.45	26.4 ± 0.75	29.7 ± 1.56	30.6 ± 0.79
	L3	33.0 ± 0.82	36.5 ± 0.79	30.0 ± 0.87	31.5 ± 0.68	25.1 ± 0.54	27.6 ± 0.79	27.0 ± 0.67	28.1 ± 1.05
	R1	33.9 ± 1.59	37.2 ± 0.24	40.7 ± 0.79	43.9 ± 0.39	29.8 ± 1.56	31.7 ± 0.65	38.8 ± 0.92	39.0 ± 0.88
	R2	30.5 ± 0.54	34.5 ± 0.74	29.0 ± 0.08	31.9 ± 1.51	29.3 ± 1.21	30.3 ± 1.24	27.7 ± 1.19	28.4 ± 0.36
	R3	33.8 ± 0.24	36.7 ± 0.70	33.0 ± 0.57	35.3 ± 1.23	32.0 ± 2.07	33.0 ± 0.93	25.9 ± 0.26	27.1 ± 1.18
Subject 2	L1	32.7 ± 1.11	39.2 ± 1.11	34.8 ± 0.43	37.3 ± 0.24	36.5 ± 0.57	40.4 ± 0.98	33.4 ± 0.63	34.2 ± 1.28
	L2	37.4 ± 0.46	39.7 ± 0.52	36.6 ± 0.79	37.5 ± 0.34	36.2 ± 0.96	36.6 ± 0.88	32.8 ± 0.40	33.9 ± 0.73
	L3	29.3 ± 1.82	36.7 ± 0.66	31.2 ± 0.85	33.3 ± 0.96	29.7 ± 0.73	30.9 ± 0.05	33.9 ± 0.42	34.8 ± 0.79
	R1	29.8 ± 0.70	32.9 ± 1.39	40.4 ± 1.13	41.1 ± 0.57	31.3 ± 1.17	33.2 ± 0.85	41.2 ± 1.63	43.4 ± 0.59
	R2	30.3 ± 0.50	33.6 ± 1.14	32.4 ± 0.49	35.8 ± 1.35	31.4 ± 1.55	34.3 ± 1.13	32.3 ± 0.36	34.1 ± 1.98
	R3	21.5 ± 0.57	25.5 ± 0.88	23.2 ± 1.01	25.8 ± 0.29	23.4 ± 0.90	26.5 ± 1.41	21.7 ± 0.34	23.3 ± 0.41
Subject 3	L1	39.6 ± 1.22	49.5 ± 1.04	40.2 ± 1.24	42.1 ± 2.05	37.1 ± 0.05	38.9 ± 0.99	37.9 ± 0.74	39.9 ± 1.59
	L2	34.3 ± 0.62	36.5 ± 0.62	32.0 ± 1.23	32.8 ± 0.50	29.5 ± 1.09	30.7 ± 0.91	30.8 ± 1.53	31.1 ± 0.05
	L3	34.4 ± 0.37	37.2 ± 0.83	26.0 ± 2.21	27.4 ± 2.65	34.3 ± 0.17	35.2 ± 0.34	32.3 ± 2.20	34.6 ± 1.11
	R1	37.5 ± 0.29	41.4 ± 1.39	35.3 ± 1.82	37.6 ± 1.10	36.9 ± 0.67	37.8 ± 0.45	37.5 ± 0.51	37.8 ± 0.87
	R2	32.8 ± 1.28	34.3 ± 0.43	28.3 ± 0.93	29.4 ± 1.55	25.7 ± 0.51	26.1 ± 0.12	26.7 ± 0.57	27.5 ± 1.52
	R3	31.4 ± 1.15	32.5 ± 0.48	30.4 ± 1.64	31.4 ± 0.99	29.9 ± 0.87	30.9 ± 0.86	30.1 ± 0.68	30.1 ± 1.16
Subject 4	L1	35.2 ± 1.33	37.5 ± 0.29	28.9 ± 1.23	29.8 ± 0.69	30.8 ± 1.00	32.8 ± 0.99	29.2 ± 0.87	30.6 ± 1.48
	L2	28.2 ± 0.34	33.8 ± 2.41	30.8 ± 1.17	33.5 ± 0.80	31.2 ± 0.92	33.2 ± 1.28	30.0 ± 1.16	30.9 ± 0.81
	L3	15.2 ± 1.03	21.0 ± 0.41	21.1 ± 0.91	21.7 ± 1.78	22.3 ± 0.36	23.4 ± 1.54	21.0 ± 0.90	21.6 ± 0.48
	R1	35.7 ± 0.50	38.8 ± 0.82	36.5 ± 0.97	37.4 ± 0.37	35.5 ± 1.10	35.6 ± 0.78	36.9 ± 0.86	37.2 ± 1.01
	R2	29.3 ± 0.97	33.1 ± 0.62	27.6 ± 0.29	29.2 ± 0.54	32.3 ± 0.45	33.3 ± 0.60	28.3 ± 1.19	30.0 ± 0.50
	R3	23.6 ± 0.95	30.5 ± 0.73	31.6 ± 0.82	32.2 ± 1.31	29.8 ± 0.29	32.0 ± 0.82	32.1 ± 0.76	34.2 ± 0.86
Subject 5	L1	30.9 ± 0.54	35.6 ± 0.05	29.4 ± 0.68	32.1 ± 0.58	31.2 ± 0.78	32.5 ± 0.42	30.0 ± 0.22	31.5 ± 0.45
	L2	27.6 ± 0.84	33.3 ± 1.51	26.4 ± 0.54	30.1 ± 0.41	25.6 ± 0.53	27.4 ± 0.45	25.4 ± 0.97	26.6 ± 0.56
	L3	24.8 ± 1.24	27.6 ± 1.88	23.6 ± 1.39	25.3 ± 1.03	25.2 ± 0.50	26.7 ± 0.62	26.3 ± 0.41	27.4 ± 0.41
	R1	34.5 ± 1.69	36.3 ± 1.16	33.1 ± 2.12	34.2 ± 0.34	35.3 ± 0.98	35.9 ± 0.66	33.1 ± 1.29	33.8 ± 0.33
	R2	27.2 ± 1.31	29.3 ± 0.59	26.9 ± 1.27	27.9 ± 0.50	28.9 ± 0.34	29.9 ± 0.63	28.7 ± 0.79	29.3 ± 0.22
	R3	24.4 ± 2.00	31.5 ± 0.54	24.7 ± 1.06	27.0 ± 0.41	28.9 ± 0.59	31.0 ± 0.36	24.3 ± 1.52	25.6 ± 0.56

From the difference of the measured values of muscular stiffness before and after overhead work activity, increasing rate of muscular stiffness is calculated for each measurement position from the following equation, and is used for evaluation:(2)

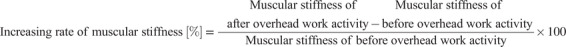



**Figure 16.**
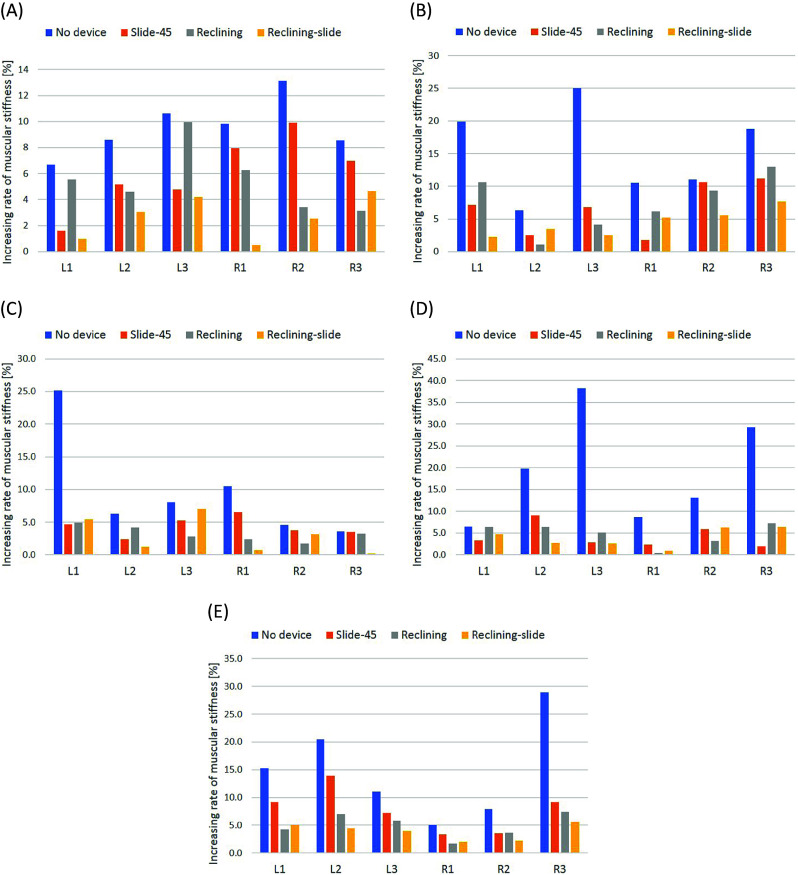
(a) Increasing rate of muscular stiffness for Subject 1. (b) Increasing rate of muscular stiffness for Subject 2. (c) Increasing rate of muscular stiffness for Subject 3. (d) Increasing rate of muscular stiffness for Subject 4. (e) Increasing rate of muscular stiffness for Subject 5.

The calculated increasing rate of muscular stiffness for each measurement position for each subject is shown in Figure 16.

For each subject, the one-sided Welch’s *t* test was conducted to the values of increasing rate of muscular stiffness in all measurement positions on each two conditions of the experiments. The results are shown in Table 9.

**Table 9.** *p*-value in each two conditions for *t* test

Condition for *t* test	Subject 1	Subject 2	Subject 3	Subject 4	Subject 5
(a) No device and device with Slide-45	.0019	.0105	.0727	.0188	.0225
(b) No device and device with Reclining	.0143	.0185	.0399	.0141	.0097
(c) No device and device with Reclining-slide	.0005	.0098	.0338	.0152	.0084
(d) Device with Slide-45 and device with Reclining	.3833	.2900	.1250	.3528	.0245
(e) Device with Slide-45 and device with Reclining-slide	.0231	.0948	.1368	.4171	.0123
(f) Device with reclining and device with Reclining-slide	.0334	.0560	.4145	.2047	.0468

From Table 8 and Figure 16, for all subjects, the increasing rate of muscular stiffness for No device is the largest and the increasing rate of muscular stiffness for Reclining-slide is comparatively small as compared with the others. In addition, from Table 9, except for condition (a) in Subject 3, a significant difference exists between “No device” and “Device with headrest” (conditions (a),(b) and (c)).

From these results, it was verified that the shoulder assist device with the headrest is effective to reduce the increasing rate of muscular stiffness for the overhead work closer to the actual work activity. Therefore, when performing an overhead work in actual work environment, reduction of a risk to develop stiff shoulders can be expected by wearing the proposed shoulder assist device with the headrest.

### 4.3. Discussion

As for the difference in types of headrests, in conditions (d), (e) and (f), except for Subject 1 and Subject 5, no significant difference exists. However, when comparing condition (b) and condition (c), *p*-values in condition (c) are smaller than those in condition (b) except for Subject 4. This result indicates that the reclining and slide-type headrest is superior to other types of headrest.

## 5. Conclusions and future works

### 5.1. Conclusions

In this article, a shoulder assist device for overhead work and a movable headrest to reduce the physical strain on the neck during the overhead work were designed and built. First, effectiveness of the built shoulder assist device and efficacy of existence of the headrest in the shoulder assist device were verified through experiments of simulating the overhead work.

Then, experiments to examine which kind of function is the most effective for the headrest were carried out under performing the overhead work activity. Based on %MVC of the integrated electromyogram measured at sternocleidomastoid muscle, the reduction effect of the physical strain on the neck was compared among the following three types of headrests; (a) Slide-type headrest, (b) Reclining-type headrest, and (c) Reclining and slide-type headrest. The experimental results showed that (c) Reclining and slide-type headrest is the most effective among these three types of headrests. In addition, the efficacy of the headrest to reduce the physical strain not only on the sternocleidomastoid muscle but also on the deltoid muscle during the overhead work activity was verified through the experiments.

Finally, a usefulness of the shoulder assist device with the headrest when performing an overhead work closer to an actual work activity was evaluated through the experiments of measuring muscular stiffness of neck and shoulder. From the experimental results, the effectiveness of the shoulder assist device with the headrest to reduce the increasing rate of muscular stiffness in the realistic overhead work was confirmed, and the superiority of (c) Reclining and slide-type headrest to other types of headrest was indicated.

From the above observations, in industrial use of the shoulder-support exoskeletons, attachment of the headrest is recommended.

### 5.2. Future works

As for future works, the followings are considered. It is necessary to perform field study in actual work environment using the built shoulder assist device with the headrest. In order to investigate a safety on the neck by wearing the proposed shoulder assist device with the headrest, dynamic analysis of cervical vertebra compressive force and cervical vertebra shear force during the overhead work activity under wearing the shoulder assist device with the headrest is worth conducting by using the musculoskeletal model software AnyBody Modeling System (AnyBody Technology Co., Ltd., Aalborg, Denmark).

## Data Availability

The data that support the findings of this study are available from the corresponding author, C. Ishii, upon reasonable request.

## A. Appendix A: Specifications

### A.1. Torsion spring used in the shoulder assist device

**Table A1.** Specifications of torsion spring used in the shoulder assist device

Manufacturer	SAMINI Co., Ltd.
Model number	33–1844
Wire diameter	2.9 mm
Material	SUS304WPB
Inner diameter	18 mm
Angle	135°
Winding number	3.125
Angle in use	41°
Spring constant	54.9 Nmm/°

### A.2. Variety motion hinge used in the headrest

**Table A2.** Specifications of variety motion hinge used in the headrest

Manufacturer	SUGATSUNE KOGYO Co.,Ltd.
Model number	HG-VH8-CL-WT
Material	Polyacetal (POM)
Mass	18 g
Opening angle	270°
Torque	Increases 0.0153 Nm every 30°
Built-in unit	Self-closing spring

### A.3. Compression spring used in the headrest

**Table A3.** Specifications of compression spring used in the headrest

Manufacturer	SAMINI Co., Ltd.
Model number	12–1232
Wire diameter	1.2 mm
Material	SUS304WPB
Inner diameter	12 mm
Free length	25 mm
Spring constant	2.39 N/mm
